# Signaling pathways in hair aging

**DOI:** 10.3389/fcell.2023.1278278

**Published:** 2023-11-16

**Authors:** Aishi Liang, Yingshan Fang, Lan Ye, Jianda Meng, Xusheng Wang, Jinsong Chen, Xuejuan Xu

**Affiliations:** ^1^ School of Pharmaceutical Sciences (Shenzhen), Sun Yat-Sen University, Shenzhen, Guangdong, China; ^2^ Endocrinology Department, First People’s Hospital of Foshan, Foshan, China

**Keywords:** hair aging, oxidative stress, hormonal disorders, inflammation, DNA damage, hair graying, hair loss

## Abstract

Hair follicle (HF) homeostasis is regulated by various signaling pathways. Disruption of such homeostasis leads to HF disorders, such as alopecia, pigment loss, and hair aging, which is causing severe health problems and aesthetic concerns. Among these disorders, hair aging is characterized by hair graying, hair loss, hair follicle miniaturization (HFM), and structural changes to the hair shaft. Hair aging occurs under physiological conditions, while premature hair aging is often associated with certain pathological conditions. Numerous investigations have been made to determine the mechanisms and explore treatments to prevent hair aging. The most well-known hypotheses about hair aging include oxidative stress, hormonal disorders, inflammation, as well as DNA damage and repair defects. Ultimately, these factors pose threats to HF cells, especially stem cells such as hair follicle stem cells, melanocyte stem cells, and mesenchymal stem cells, which hamper hair regeneration and pigmentation. Here, we summarize previous studies investigating the above mechanisms and the existing therapeutic methods for hair aging. We also provide insights into hair aging research and discuss the limitations and outlook.

## Introduction

**FIGURE 1:**
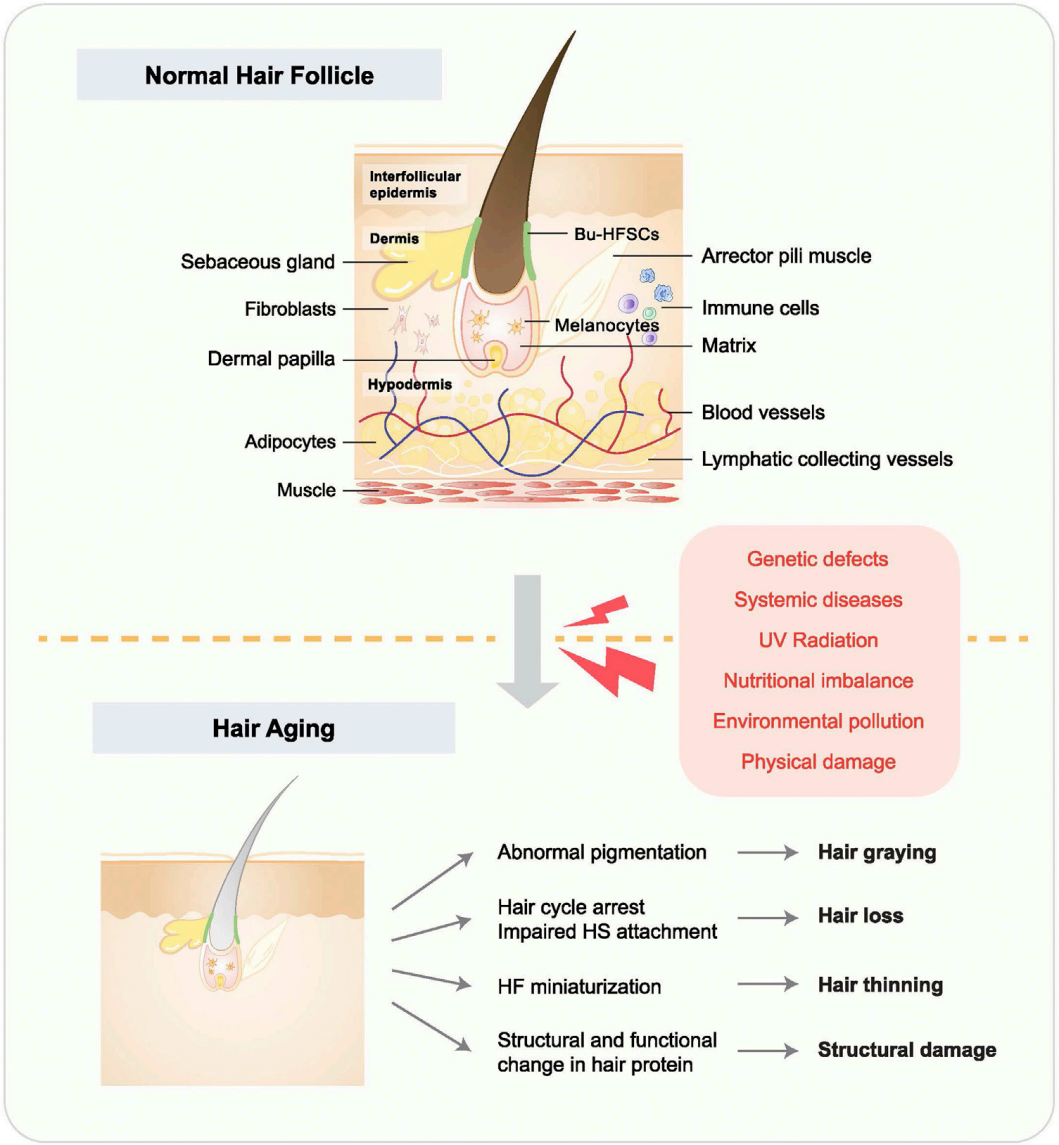
Hair follicle structure and hair aging phenotypes. HF accommodates multiple tissues and cells, and their malfunction leads to hair aging. Causes of hair aging are listed in the red box. Hair aging is largely manifested by hair graying, hair loss, hair thinning, and hair shaft structural change.

Hair follicles (HFs) are constituted by different cell types, including hair follicle stem cells (HFSCs), non-HFSC epithelial cells, immune cells, neurons, mesenchymal cells, adipocytes, and melanocytes. Other structures, such as sebaceous glands (SGs), blood vasculature, and arrector pili muscle (APM), are also important HF components (Lei et al., 2021) (Figure 1). Generally, HF status depends on the hair cycle, which can be roughly divided into three stages, including anagen (the growing phase), catagen (the transition phase), and telogen (the resting phase). These phases are modulated by genes, age, microenvironment, diet, and psychological factors (Alonso and Fuchs, 2006). HF homeostasis is disrupted due to aging, gene mutations, nutritional imbalance, hormonal dysregulation, the inflammatory microenvironment, etc., which will lead to various HF disorders such as hair aging (Palmer et al., 2020). Although hair-related diseases are not life-threatening, they can significantly influence people’s social activities and psychological wellbeing (Mercke et al., 2000; Cotsarelis and Millar, 2001). Among these disorders, hair aging is manifested by hair graying, hair loss, hair thinning, hair follicle miniaturization (HFM), structural changes, lipid composition change, and curvature in the hair fiber (Trüeb, 2006). There are multiple causes of hair aging, including genetic defects, systemic diseases, ultraviolet (UV) radiation, nutritional imbalance, environmental pollution, and physical damage (D’Orazio et al., 2013).

Hair aging is often accompanied by hair graying, hair loss, and hair thinning (Figure 1). The hair pigmentation process starts with melanocyte stem cells (McSCs), which differentiate into melanocytes to produce pigmentation units. During anagen, melanocytes go through mitosis and are activated, manifested by increasing dendricity. Through the dendrites, they can transfer melanosomes, which contain melanin (Van Neste and Tobin, 2004; Tobin, 2008). Hair graying happens when the pigmentation process is disrupted (Nishimura et al., 2005). For example, it was recently reported that McSCs could switch between transit-amplifying status and quiescence status and reside in a dynamic niche, indicating a potential role of McSC mobility in regulating cell stemness and hair graying (Sun et al., 2023). Hair loss, however, is mostly related to HFSC dysfunction and depletion. Physiologically, HFSCs are activated at anagen and stay quiescent at telogen. Whereas, in alopecia, HFSCs are depleted or remain in a quiescent status, leading to irreversible or reversible hair loss, respectively (Hu X.-M. et al., 2021). HFSCs are regulated by intrinsic and extrinsic cues, such as Wnt and bone morphogenetic protein (BMP) signaling, as well as skin wounding (Kandyba et al., 2013; Lee et al., 2021). Hair thinning can be a transitional status before hair loss, frequently occurring with HFM, which is manifested by the reduction of the diameter of HFs and hair shaft (Anastassakis, 2022).

**FIGURE 2:**
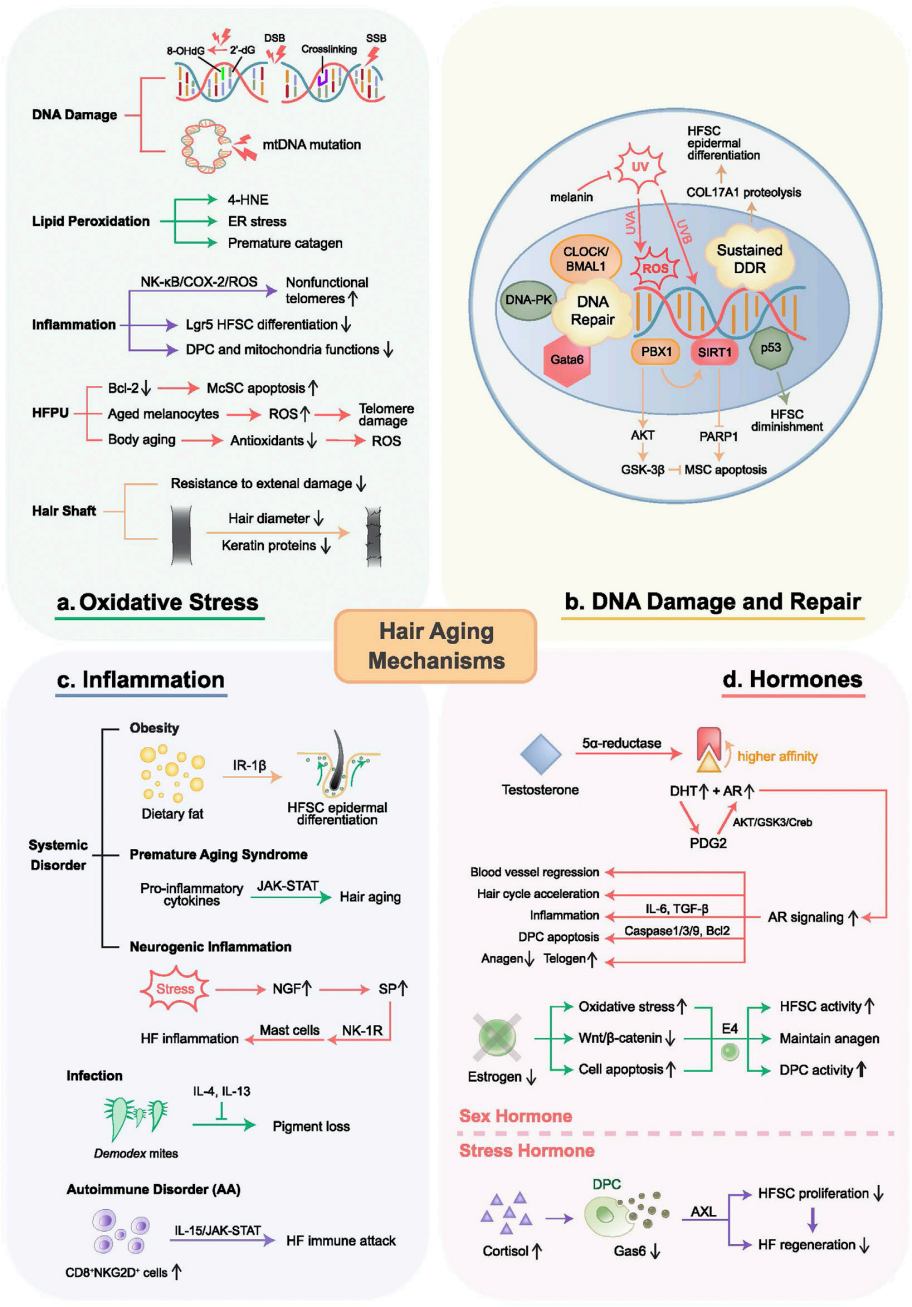
Mechanisms of hair aging. Proposed mechanisms of hair aging include oxidative stress, inflammation, DNA damage and repair, as well as hormone disorders. **(A)** Oxidative stress causes DNA damage, protein modifications, lipid oxidation, chronic inflammation, and HFPU damage. **(B)** DNA damage can be caused by UV and genetic defects such as p53 and p63, leading to HF-residing stem cell dysfunctions through the PBX1/AKT/GSK-3β and PBX1/SIRT1/PARP1 axes. Sustained DNA damage-induced DDR results in HFSC epidermal differentiation. Cell resistance to DNA damage is determined by DNA repair capacity, regulated by CLOCK/BMAL1, DNA-PK, and Gata6. **(C)** Inflammation results from systemic disorders, infections, and autoimmune disorders, mediated by inflammatory signaling and immune cell attack. **(D)** Androgens activate AR signaling, upregulating genes related to hair growth inhibition, apoptosis, vascular regression, DPC aging, and hair aging, exacerbated by PGD_2_. Conversely, estrogens maintain the hair cycle, protect against oxidative stress, and promote follicle health. E4 induces DP fibroblasts, increasing anagen follicles and CD34^+^ cells in the outer root sheath to delay HF aging. Chronic stress accelerates hair loss and follicle aging through the cortisol-Gas6-AXL axis. DSB, double-strand break; SSB, single-strand break.

Numerous theories exist about the primary mechanism underlying hair aging. The most well-known one is the thesis of oxidative stress, which accounts for multiple kinds of cell dysfunction such as mitochondrial damage and upregulated inflammatory signaling (Williams et al., 2020). Additionally, extensive research is being done on other possibilities, including hormone-induced premature hair aging, inflammation-predominant hair aging, and DNA damage-driven hair aging (Figure 2). The following sections will give detailed depictions of these concepts. In this review, we try to outline and update the signaling pathway underlying these hair aging hypotheses and provide insights into the current progress and limitations of hair aging research.

## Oxidative stress-induced hair aging

The Free Radical Hypothesis was first posited in the 1950s and is widely used to elucidate the mechanisms underlying the aging process (Arck et al., 2006). It is stated that aging is caused by the built-up redox damage to the biomolecules due to the imbalance in the oxidation-reduction processes (Arck et al., 2006). As an evolved version of the theory, mitochondrial dysfunction, has been highlighted as the major contributor of oxidative stress. It is stated that oxidative stress may lead to mitochondrial DNA (mtDNA) mutations and deletions (Ziada et al., 2020). Oxidative damage is mainly created by excessive reactive oxygen species (ROS), primarily produced during metabolic process (Liu et al., 2022).

ROS comprises a group of highly reactive oxygen molecules or free radicals, mostly generated in mitochondria during oxidative phosphorylation, such as superoxide anion (O2•^−^), hydrogen peroxide (H_2_O_2_), and hydroxyl radical (•OH) (Balaban et al., 2005). External factors such as UV radiation, ionizing radiation, and environmental toxins may also increase levels of ROS (Tirichen et al., 2021). Although most cells have certain capabilities to clear up ROS, oxidative damage can still occur when the antioxidant systems are overwhelmed by the adverse impacts of elevated ROS levels, leading to premature aging.

Current research has demonstrated that mechanisms underlying oxidative damage in HFs mainly involve ROS-induced DNA damage, impaired antioxidant enzyme activity, oxidative modification of hair proteins, induction of chronic inflammation, and damage to hair follicle pigmentary unit (HFPU) (Trüeb, 2021).

ROS can damage DNA through various mechanisms. ROS, particularly hydroxyl radicals (•OH), can directly react with the DNA molecule’s bases, causing base oxidation, deoxyribose damage, and DNA strand breaks. For instance, ROS can cause base oxidation including 8-oxoguanine and 8-hydroxy-2′-deoxyguanosine (8-OHdG), as being pivotal markers for measuring the effect of endogenous oxidative damage to DNA. These modifications disrupt normal base pairing, interfering with DNA replication and transcription (Tarng et al., 2000). Moreover, recent research has indicated that ROS might reduce the fidelity of mitochondrial polymerase, potentially leading to an increase in somatic transition mutations (Anderson et al., 2020). In addition to that, another role of ROS is to serve as signaling molecules and affect the generation of mitochondria, further contributing to the spread of mtDNA mutations (Napoli et al., 2013; Martins et al., 2021).

Moreover, ROS can oxidize unsaturated fatty acids, thus establishing structural and functional changes in the cell membrane, involving loss of selective permeability, unusual opening of ion channels, etc. For example, 4-hydroxynonenal (4-HNE) is a reactive aldehyde generated from the reaction between ROS and polyunsaturated fatty acids in lipids (Barrera, 2012; Juan et al., 2021). 4-HNE forms adducts with protein amino acid residues (mainly cysteine, histidine, lysine) through Michael addition and Schiff base formation, leading to protein crosslinking and aggregation (Su et al., 2019). Additionally, 4-HNE can further activate the Keap1/Nrf2/ARE pathway, consequently promoting oxidative stress. In addition, lipid peroxidation can cause endoplasmic reticulum (ER) stress, leading to protein misfolding and aggregation (Milkovic et al., 2023). Specifically, Naito et al. found that topical application of linolein hydroperoxides, a type of lipid peroxide, induced premature catagen phase onset in murine hair cycles (Csala et al., 2015). Furthermore, with age, the activities of superoxide dismutase (SOD) and catalase (CAT) are diminished, and their ability to clear the ROS is impaired, thereby increasing susceptibility to oxidative damage (Naito et al., 2008).

Oxidative stress can also trigger oxidative modifications in hair proteins, such as keratin (Rybka et al., 2013). The concomitant structural and functional changes in the proteins can result in hair shaft aging. Studies have found that aged hair has a reduced diameter and decreased keratin-associated proteins (KAPs) (Trüeb, 2009). Additionally, oxidative stress can lead to lipid peroxidation in the hair shaft, particularly peroxidation of cholesterol, thus abolishing its abilities to protect the hair shaft from chemical damage, to prevent hair softening, to reduce diameter shrinkage, and to preserve hair shine (Giesen et al., 2011).

Recent studies have demonstrated that ROS can upregulate the transcription of various proinflammatory mediators and impair tissue regeneration (Brosche et al., 2001; Shafiq et al., 2021). For example, the diminished tissue regenerative potential and accelerated aging in nfkb1^−/−^ mice were shown to be associated with chronic activation of NF-κB/COX-2/ROS axis by inflammation, which resulted in the accumulation of nonfunctional telomeres (Jurk et al., 2014; Mittal et al., 2014). Additionally, Yang, et al. revealed that wound-induced IL-1α facilitates the differentiation of leucine-rich repeat G protein-coupled receptor 5 (Lgr5) HFSCs by decreasing inflammatory cell infiltration and ROS levels, thereby promoting the neogenesis of aged hair follicles (Zhang et al., 2016). Furthermore, it was documented that Argan press cake can protect DPCs from H_2_O_2_-induced oxidative stress by attenuating inflammation and downregulating ROS-associated gene expression, thus restoring cellular viability and mitochondrial function. However, there is no direct evidence that ROS-induced inflammation accelerates hair aging (Yang et al., 2023). While studies have demonstrated that restraining ROS and inflammation can facilitate HF regeneration, the precise mechanisms by which ROS and inflammation directly impact HF aging or impede its regeneration remain to be fully elucidated.

Furthermore, hair aging is commonly manifested by pigment loss due to dysfunction of HFPU, which is highly sensitive to ROS. Bcl-2, an anti-apoptotic protein, maintains the redox balance within the follicular stem cell niche. It is reported that depletion of Bcl-2 exacerbates oxidative stress, inducing McSCs selective apoptosis and consequently hampering the repopulation of melanocytes in anagen follicles (Bejaoui et al., 2021). Research also indicates that with age, the clearance capacity of ROS declines, resulting in ROS accumulation that damages HFPU, leading to hair graying (Seiberg, 2013). Consistent with Harman’s original free radical aging thesis, another hypothesis suggests that melanin can act as an antioxidant, but the process of melanin synthesis itself generates a substantial amount of ROS (Arck et al., 2006). This underscores the significance of maintaining a harmonious redox balance in young and healthy HF, signifying the importance of properly-working ROS clearance machinery. Additionally, similar to the skin aging induced by senescent epidermal melanocytes, it is believed that HF melanocytes are likely to serve as cellular stress sensors, and paracrine signals from aging melanocytes in the HFPU increase ROS production and induce telomere damage, accelerating HFPU aging (O’Sullivan et al., 2021).

## Hormone accelerates hair aging

Androgens, such as testosterone and DHT, are primarily secreted by the adrenal glands, the testicles in the male reproductive system, and the ovaries in the female reproductive system (Stone et al., 2021). Testosterone and DHT influence HFs by binding to androgen receptors (AR) within the follicular cells. Testosterone can be converted into DHT by 5α-reductase, with a higher affinity of approximately 5–10 folds for ARs. In the balding scalp of male pattern hair loss (MPHL) patients, 5α-reductase and DHT were found to increase abnormally, thus overactivating the AR signaling (Handelsman and Feingold, 2000). Consequently, AR signaling upregulated the expression of dikkopf-related protein 1 (DKK-1), an inhibitor of WNT signaling, IL-6, and transforming growth factor β (TGF-β). As a result, hair growth and the telogen-to-anagen transition were inhibited (Ustuner, 2013). Besides, microvascular vessel aggregation around DP is crucial for anagen initiation (Cerut et al., 2018). Recently, Deng et al. reported that AR-induced blood vessel regression around DP in the initial stage of HFM might be one of the causes of pattern hair loss (PHL). Specifically, when ARs in the dermal papilla cells (DPCs) sensed the increase of androgen, they facilitated DKK-1 and TGF-β expression as well as antagonized Wnt/β-catenin pathways to suppress hair shaft growth (Stenn and Paus, 2001). TGF-β further shrank the blood vessels, thus synergistically inhibiting hair growth and promoting the process of HFM (Deng et al., 2022). Interestingly, Jung et al. reported that DHT could promote the accumulation of mitochondrial ROS (mtROS) in DPCs by activating membrane androgen receptor (mAR) signaling. The mtROS accumulation led to the formation of mitochondria-associated ER membranes (MAM) and thus induced mitochondrial Ca^2+^ accumulation through the interactions between VDAC on mitochondria and IP3R1 on ER, ultimately resulting in increased DPC aging. Also, they found that DPC aging could be mitigated by Cyanidin 3-O-arabinoside through its antioxidative effects and the inhibition of the p38 signaling pathways in an androgenetic alopecia (AGA) mouse model (Rushton et al., 2022).

Moreover, the research conducted by Garza et al. showed that testosterone production was associated with prostaglandin D (PGD) (J et al., 2022). PGD synthase was found to be overexpressed in the alopecia area, and evidence supports that surplus PGD_2_ can induce precocious catagen as well as shortened anagen (J et al., 2022). Jeong et al. also reported the specific mechanism of PGD_2_ interacting with AR signaling (Garza et al., 2012). Particularly, PGD_2_ interacted with the DP_2_ receptor to upregulate AR expression, thus increasing its downstream effectors such as TGF-β1 and LEF1. PGD_2_ also induces the expression of cell apoptosis genes such as caspase-3 and caspase-9. Additionally, PGD_2_ could activate the AKT/(glycogen synthase kinase-3β (GSK-3β)/Creb axis to further enhance AR signaling within hDPCs (Garza et al., 2012).

In short, excessive androgens can induce the HF transition from the anagen to the catagen and telogen, effectively shortening the duration of the anagen. This premature entry into the resting phases leads to hair shedding, thereby contributing to HF aging (Jeong et al., 2018).

Estrogens are also important in hair cycle regulation. Studies have demonstrated that postmenopausal women are more likely to suffer from female pattern hair loss (FPHL) because of reduced ovary-derived estrogen. Conversely, elevated estrogen levels occur with increased anagen HFs, particularly during pregnancy (Ho et al., 2022). Estrogens are also important in maintaining normal HF functions (Hasan et al., 2022). Estrogens can activate the Wnt/β-catenin signaling to maintain HFSC proliferation and differentiation, promoting healthy hair growth (Chen et al., 2020). Estrogens also protect HFs from oxidative damage by regulating the expression of antioxidant enzymes, which slows down HF aging (Choi, 2020). Additionally, estrogens can inhibit the apoptosis pathway within HF cells by affecting the expression and activity of Bcl-2 family proteins and regulating the levels of apoptotic factors (Serruya and Maor, 2021). Furthermore, recent research has revealed that treatment with estetrol (E4), an endogenous estrogen produced by the fetal liver, could significantly increase the number of anagen HFs with enhanced hair matrix keratinocyte proliferation. Moreover, E4 treatment also stimulated DP fibroblast inductivity, as evidenced by an upregulation of versican expression and alkaline phosphatase activity in the DP, particularly in anagen HFs. E4 also promoted the generation of stem cell progeny by increasing the percentage of CD34^+^ cells (a marker for diverse progenitors) in the outer root sheath (ORS) (Hu et al., 2012). Thus, E4 has high potential for hair loss treatment.

Additionally, chronic stress can accelerate HF aging and hair loss by negatively impacting the activity and proliferation of HFSCs. Corticosterone (equal to cortisol in rodents), a major stress hormone, prevents HFSCs from entering the anagen, mainly through the corticosterone/Gas6/AXL axis. Mechanistically, cortisol binds to glucocorticoid receptors (GR), resulting in Gas6 suppression. Thereby, the AXL receptors cannot be sufficiently stimulated, which aborts HFSC activation and contributes to accelerated HF aging (Gerard et al., 2023).

## Inflammation-predominant hair aging

Inflammation is another factor that causes hair to age. Inflammation is associated with cell death and compromised cell functions. It is reported that inflammation-induced ROS could impair mitochondrial functions, melanosome transfer, and melanocyte migration, leading to hair graying (Choi et al., 2021). In addition, HFSC aging is also largely regulated by inflammatory signals (Ji et al., 2017; Jo et al., 2018), which are closely correlated to hair thinning and hair loss. Generally, HF inflammation occurs in the context of systemic disorders, autoimmune disorders, and infections.

Hair aging happens with systemic disorders such as obesity, progeria diseases, and psychological stress. Morinaga et al. investigated the role of HFSCs in obesity-induced hair loss (Castellana et al., 2014). They discovered that high-fat diet (HFD) led to HFSC epidermal differentiation, mimicking aging HFs, which could be attributable to IL-1 inflammatory signaling. While local IL-1β administration substantially increased Cox2 expression in HFSCs from old mice, Cox2 was not upregulated in HFSCs from young mice, suggesting an age-dependent decrease in resistance to acute inflammatory signals. These results show that obesity-induced inflammatory signals account for premature hair loss, and the effects are age-dependent. Besides, Bedja et al. also observed hair graying and hair loss in atherosclerosis (ApoE^−/−^) mice supplied with HFD with high cholesterol, together with neutrophil infiltration and increased tumor necrosis factor-stimulated gene-6 (TSG-6) within the skin (Morinaga et al., 2021). A survey conducted by Shin et al. further demonstrated a strong association between obesity and premature hair graying (Bedja et al., 2018).

Systemic progeria diseases are manifested by the senescence of hair, including Hutchinson-Gilford progeria syndrome (HGPS) and Werner syndrome, and they often occur with augmented inflammation (Shin et al., 2015). For example, pro-inflammatory cytokines were found to mediate JAK-STAT signaling overactivation in HGPS (Trüeb, 2005). Other research further confirms the role of JAK-STAT in hair loss. Wang et al. found that JAK-STAT5 activation could induce HFSC quiescence as a downstream target of Oncostatin M (OSM), which was secreted by TREM2^+^ macrophages (Arnold and Djabali, 2019). Consistently, administration of baricitinib, a JAK inhibitor, was sufficient to blunt cell senescence by downregulating pro-inflammatory signals including IL-6, CXCL8, IL-4, and TNF-ɑ. Notably, baricitinib has recently been approved by the FDA as a treatment for alopecia areata (AA), an autoimmune disorder with phenotypes of hair loss with preserved HFs (Wang et al., 2019). AA is caused by immune privilege breakdown with the aggregation of NK cells, CD4^+^ T cells, and CD8^+^ T cells around HFs (Freitas et al., 2023). Among these immune cells, CD8^+^NKG2D^+^ cells play a central role in pathogenesis, whose survival depends on the IL-15/JAK-STAT pathway. Consistently, baricitinib, as a JAK inhibitor, was shown to be potent enough to alleviate AA and normalize AA signatures, including IFN-γ pathway as well as cytotoxic T lymphocyte (CTL) (Pratt et al., 2017). Interestingly, several studies have shown that hair aging is also associated with other autoimmune disorders, such as pernicious anemia and autoimmune thyroid disease (Trüeb, 2006).

Premature hair loss and graying can also result from systemic stress and the following neurogenic inflammation, which is generated by nerve activation and can lead to neuropeptide release, connecting the brain and the skin (Jabbari et al., 2015). In contrast to the systemic stress response commonly observed in other organs, neuropeptides can be produced locally in HFs to trigger immune cell infiltration, which causes cytokine-mediated inflammation in HFs (Matsuda et al., 2019). For example, it is indicated that stress-induced nerve growth factor (NGF) promoted substance P (SP) synthesis and release from the nerve ends in HFs. NGF and SP further stimulated neurogenic inflammation, primarily via mast cells (O’Sullivan et al., 2022). SP causes inflammation in HFs mainly through interaction with the neurokinin-1 receptor (NK-1R). Siebenhaar et al. illustrated that the SP/NK-1R could cause mast cell degranulation as well as activate CD8^+^ T cells in HFs of AA mouse models, indicating profound neurogenic inflammation (Paus et al., 2014). These results suggest that the SP/NK-1R/mast cell pathway plays an important part in psychoemotional stress (Siebenhaar et al., 2007).

Hair aging also happens as a result of infections. Research has shown an age-related microbiome change in scalp skin. Utilizing bacterial 16S rRNA sequencing, they found that aged skin accommodated a greater variety of microbiomes than young skin, and the microbiome exhibited significant proportional change (Liu et al., 2013). The microbiome discrepancy indicates its potential role in hair aging. It has already been demonstrated that HF-residing bacteria are associated with several scalp diseases, including folliculitis decalvans, AA, and AGA (Tirichen et al., 2021). However, how these microbiomes correlate to the hair aging process is still under investigation. Besides bacteria and fungi, microorganisms such as *Demodex* mites also contribute to hair aging. In common situations, *Demodex* remains at a low level, with increasing infestation in aging skin (Shibagaki et al., 2017). Upon *Demodex* infection, Ricardo-Gonzalez et al. observed substantial pigment loss and revealed a significant role of type 2 immunity in defense against *Demodex*, which was mediated by IL-4 and IL-13 (Lacey et al., 2011). Administration of IL-4 and IL-13 further inhibited HFSC proliferation as well as hair growth, suggesting that *Demodex-*induced hair aging is mediated through inflammatory signaling.

## Hair aging driven by DNA damage and repair defects

Another major triggering factor for cell dysfunction and tissue aging is DNA damage. DNA damage induces aging not only by changing the genetic profiles but also by altering the chromatin structure, the length of telomeres, mitochondrial functions, and proteostasis (Vermeij et al., 2014; Ricardo-Gonzalez et al., 2022). Ultimately, DNA damage accumulation is associated with cell transformation, senescence, and apoptosis (Best, 2009; Schumach et al., 2021). This also applies to the hair aging process, with a strong impact on HF-residing stem cells, including HFSCs, McSCs, and mesenchymal stem cells (MSCs). Despite their resistance to DNA damage under physiological conditions, their DNA repair capacity decreases with age (Maynard et al., 2015). Therefore, the influence on hair aging depends on the extent of DNA damage as well as the ability to repair. Here, we introduce multiple causes for DNA damage and how they lead to stem cell dysfunction in HF. We also highlight the role of the DNA repair system in preventing hair aging.

DNA damage in HF cells can be triggered by several factors, such as ultraviolet (UV) radiation, genetic defects, and chemotherapies (Schuler and Rübe, 2013). Photoaging happens through direct or indirect effects, mediated by UVB and UVA, respectively. UVB promotes direct DNA damage by rearranging nucleotides and generating photoproducts such as cyclobutane dimers, while UVA produces ROS to damage mitochondria and DNA (D’Orazio et al., 2013). Zhai et al. indicated that UVA radiation reduced the number of HFSCs and melanocytes in HFs, attributable to HFM and hair graying noted in mice. UVB, on the other hand, was sufficient to make histological alterations to HFs, with thickened epidermis and hyperplastic SGs (Yousefzadeh et al., 2021). It is believed that UV-induced DNA damage can be prevented by melanin, which absorbs UV penetrating through, emphasizing the role of McSCs and melanocytes in photoaging prevention (Zhai et al., 2021).

Genetic dysfunction accounts for hair aging as well, such as p53 and p63 mutations. The p53 transcription factor inhibits cell proliferation and induces cell death to accelerate the aging process, and it is commonly mutated in human cancers (García-Cao et al., 2002; Panich et al., 2016; Gritsenko et al., 2017). The introduction of T21D and S23D mutations in p53 mimics its phosphorylated state, inducing an alteration in Mdm2 binding region, a negative regulator of p53, thus magnifying p53 downstream effects (Rufini et al., 2013; Kim et al., 2014). p53 mutants displayed hair cycle arrest as well as declined SG activity and reduced thickness of subcutaneous adipose, which might contribute to the hair aging process (Rufini et al., 2013). In another study, p53 signaling was overactivated by Mdm2 deletion, resulting in significant hair loss and HFSC diminishment in aged mice but not in young mice. Concomitantly, wound healing capacity was impaired and hair regrowth was delayed, especially in old mice (Liu et al., 2010). Despite the accelerated aging induced by p53, its homolog p63 is able to prevent aging (Gannon et al., 2011). Su et al. demonstrated that the deletion of TAp63, an isoform of p63, triggered premature hair aging and HF loss with enhanced SA-β-gal signals, a cellular senescent marker. Interestingly, massive DNA damage was detected in TAp63^−/−^ mice, suggesting that TAp63 might delay aging by maintaining genome stability (Craig et al., 2010).

Other causes of DNA damage in HFs include irradiation, medical treatment, and environmental pollutants. Kim et al. uncovered the mechanisms for alkylating chemotherapy-induced permanent hair loss (Su et al., 2009). They revealed that HFSCs went through rapid proliferation after treatment with busulfan followed by cyclophosphamide, which impaired HFSCs’ resistance to DNA damage and depleted the HFSC pool. In addition to that, Titova et al. explained that DNA damage in the skin could also be caused by terahertz (THz) radiation, widely used in diagnostic imaging and security screening (Kim et al., 2019). Toxins such as patulin and ochratoxin A were found to impact genome stability in the skin as well (Saxena et al., 2009; Titova et al., 2013).

Mentioned above are the causes of DNA damage in HF cells, which often result in stem cell dysfunction to induce hair aging. For example, hair thinning and hair loss in aged mice occur with sustained DNA damage response (DDR) in aging stem cells. Particularly, DDR in aging HFSCs caused type XVII collagen (COL17A1) proteolysis, promoting epidermal differentiation of HFSCs and diminishing their stemness (Kumar et al., 2012). In addition to HFSCs, hair follicle mesenchymal stem cells (HF-MSCs) are also an important player in HF regeneration and wound healing, and their senescence might lead to hair aging. Wang et al. indicated that DNA damage-induced HF-MSC apoptosis could be rescued by PBX homeobox 1 (PBX1) overexpression (Matsumura et al., 2016). Further studies demonstrated that PBX1 alleviated HF-MSC apoptosis through a PBX1/AKT/GSK axis (Wang et al., 2021). In another study, PBX1 upregulation was accompanied by increased SIRT1 as well as decreased PARP1 expression, and DNA damage was also alleviated. These results suggest a PBX1/SIRT/PARP1 axis in DNA damage reduction and HF-MSC maintenance (Jiang et al., 2019). Additional study has revealed that shortened telomeres broadly affected stem cells within the epidermis, which impaired the epidermis as well as HF formation (Wang et al., 2023). Telomere shortening can happen from DNA damage and is proposed as one of the major aging mechanisms, restricting cell division and changing gene expression (Vermeij et al., 2014). Mechanically, telomere dysfunction reduces the number of epidermal stem cells and impedes their specification and differentiation through a BMP/pSmad/P63 axis, contributing to skin atrophy and hair loss.

Aside from DNA damage, defects in DNA repair also account for hair aging. Gata6 is a transcriptional factor expressed in HF progenitors. Ablation of Gata6 in mice exhibited cell cycle arrest in keratinocytes and impaired progenitor differentiation, accompanied by significant DNA damage marked by γ-H2AX and increased apoptosis marked by caspase3 (Liu et al., 2019). These results suggest the anti-aging role of Gata6 in HFs. Additionally, circadian clock proteins are involved in DNA repair, which can be transcriptionally activated by the CLOCK/BMAL1 heterodimer complex. Bmal1 deficiency facilitated ROS accumulation and thus DNA damage to HF-residing stem cells in aged mice, promoting hair aging (Wang et al., 2017). Furthermore, Sotiropoulou et al. found that DNA-PK could accelerate non-homologous end-joining repair of double-strand DNA breaks, increasing bulge-SCs’ resistance to DNA damage-induced apoptosis (Geyfman and Andersen, 2010). Collectively, these studies highlight the role of DNA repair in preventing hair aging.

## Treatment

### Drug treatment

With the increasing social emphasis on hair research, extensive investigations into hair loss treatment have been conducted over several decades. However, in the context of hair aging, the existing treatment methods remain significantly limited. Currently, topically applied minoxidil and orally administered finasteride are the two most commonly used drugs approved by the FDA for the treatment of hair loss (Sotiropoulou et al., 2010; Wang et al., 2019).

#### Minoxidil

Initially, minoxidil acts as an oral antihypertensive medication as an activator of potassium (K^+^) ion channels (Devjani et al., 2023). Although the exact manner in which topical minoxidil promotes hair growth remains unclear, current studies indicate that it most likely works through vasodilation, anti-inflammatory action, induction of Wnt/β-catenin signaling, resistance to androgens, and modulation of the hair cycle (Campese, 1981). The most common formulations include 2% solution, 5% solution, foam, and spray in clinical treatment (Gupta et al., 2022). Many patients experienced adverse effects such as skin itching or allergies that were associated with PEG in the solution formulations (Kovacevic et al., 2020). Additionally, only about 60% of the male AGA patients respond to topical minoxidil treatment in practical use (Friedman et al., 2002). Clinically, patients’ suitability for minoxidil can be determined by the sulfotransferase assay (FSA) (Goren et al., 2014). However, the specific role of FSA in minoxidil treatment warrants further investigation due to the high variability of sulfotransferase expression.

#### Finasteride

Another FDA-approved drug for AGA treatment is finasteride. It selectively inhibits types II and III 5α-reductase isoenzymes and consequently hampers the conversion of testosterone into DHT, which has a stronger affinity for AR, thereby downregulating AR signaling (McCoy et al., 2019). Nonetheless, finasteride cannot sufficiently block type I 5α-reductase isoenzyme, DHT conversion does not stop completely, and when medication is stopped, DHT levels are restored in the second week (Rittmaster, 1994). Moreover, within 12 m following the discontinuation of treatment, the hair regrowth will be reversed (Rittmaster, 1994). Furthermore, clinical observations suggest that finasteride exhibits systemic effects, which potentially increase the risks of prostate cancer and sexual disorders in male patients (Lee et al., 2018), highlighting the need to develop a topical treatment approach (Mysore, 2012).

### Non-drug treatment

The constraints of these two drugs have prompted people to explore new therapies. For example, platelet-rich plasma (PRP) and low-level light therapy (LLLT) have emerged as alternative approaches for hair regeneration.

#### Platelet-rich plasma (PRP)

PRP is separated from patients’ whole blood and contains high levels of platelets, approximately 5–6 times more platelets than normal (Gupta and Talukder, 2022). Many growth factors and cytokines related to hair growth can be emitted by platelets in PRP, including PDGF, VEGF, EGF, and TGF-β (Dashore et al., 2021). Additionally, PRP can induce DPC proliferation by activating the ERK1/2, PI3K/AKT, and Wnt/β-catenin signaling pathways (Straum, 2020). Also, due to its few side effects as an autologous plasma product and promising treatment efficacy, PRP serves as an effective therapy alone or in combination with other treatments and hair transplantation (Xiao et al., 2019). Many studies have proven the effectiveness of PRP in different forms of alopecia, such as AGA, FPHL, and AA. Whereas, its mechanisms still need further clarification (Garg and Manchanda, 2017; Everts et al., 2020; Abdin et al., 2022).

#### Low-level light therapy (LLLT)

LLLT utilizes lasers (600–1,100 nm) to promote hair growth and increase the proportion of anagen HFs, without an invasive procedure (Stevens and Khetarpal, 2018). Until 2023, more than 47 LLLT devices have been designed and approved by the FDA, and there have been multiple clinical studies demonstrating the efficacy of LLLT, which is even more potent than the FDA-approved drugs in some cases (Farivar et al., 2014; Pillai and Mysore, 2021). The convenience, high patient compliance, good efficacy, and minimal side effects have made it a highly anticipated therapeutic approach (Lueangarun et al., 2021). Current studies suggest that LLLT may promote hair growth by increasing adenosine triphosphate (ATP) production, regulating ROS, and reducing inflammatory PGE_2_ through photobiomodulation (Farivar et al., 2014; Stevens and Khetarpal, 2018; Taylor et al., 2020).

Nonetheless, there are numerous unresolved issues with this therapy. The exact mechanisms for promoting hair growth remain unclear, and there is a significant disparity in its efficacy between humans and animals. The challenge also lies in the standardization of treatment protocols, which should be based on individual differences such as hair lengths, colors, and skin tones (Sakurai et al., 2000).

### Stem cell therapies

Much effort has been made to reverse hair aging by boosting stem cell activity. Stem cell transplantation, hair follicle organoids, and stem cell-derived exosomes have gained attention as potential therapies to rejuvenate the HFs (Anastassakis and Anastassakis, 2023).

#### Stem cell transplantation therapy

Stem cells possess intrinsic features such as self-renewal, mobility, anti-inflammatory properties, and immunity that promote repair and regeneration in aging tissues. Stem cell therapies for hair aging mainly include MSC treatment due to its pluripotent regenerative potential (Egger et al., 2020). Depending on the origin, these MSCs can be categorized into adipose tissue-derived MSCs (AD-MSCs), bone marrow-derived MSCs (BM-MSCs), and perinatal MSCs, etc. (Egger et al., 2020). Some studies have indicated that growth factors secreted by AD-MSCs can activate epidermal stem cells and DPCs. Furthermore, AD-MSCs display anti-inflammatory effects through cell-cell interactions as well as modulation of PGE_2_ and leukemia inhibitory factors. Clinically, MSC transplantation in AGA patients has achieved favorable therapeutic outcomes. However, the limits on the acquisition and cell quantity of AD-MSCs and BM-MSCs remain a bottleneck of this therapeutic approach (Shimizu et al., 2022).

#### Hair follicle organoids

Derived from embryonic stem cells (ESCs) and induced pluripotent stem cells (iPSCs), hair follicle organoids are shown to promote hair regeneration (Lee et al., 2022; Salhab et al., 2022). Within hair follicle organoids, there are stratified layers of epidermis and dermis, as well as appendages such as neurons, SG, and DP (Lee et al., 2022). Also, hair follicle organoids exhibit higher hair production capacity compared to directly cultured HFs (Lee et al., 2020). Additionally, iPSC-derived hair follicle organoids significantly mitigate ethical concerns related to ESC treatment (Sun et al., 2021). However, current hair follicle organoid cultivation protocols are not able to induce sweat glands, immune cells, blood vessels and APM. Moreover, the HFs induced by current methods resemble vellus hair rather than thick terminal hair (Salhab et al., 2022). This issue might be tackled by optimizing the organoid culture protocol. According to the current method, the DP structure is derived from cranial neural crest (CNC) cells, while DP within the human scalp HF originates from mesodermal cells (Lee et al., 2022). Since DP is critical in controlling the duration of anagen, which distinguish the terminal hair and the vellus hair (O’Sullivan et al., 2021), establishing a method to culture ectoderm and mesoderm-derived hair follicle organoid might help break the bottleneck. Furthermore, preventing post-transplantation carcinogenesis is a critical concern in stem cell transplantation (Vatanashevanopakorn and Sartyoungkul, 2023).

#### Stem cell-derived exosomes (SCD-Exos)

Exosomes are tiny membrane-bound vehicles, about 40–160 nm in diameter, released from cells via the endosomal pathway. They serve as crucial messengers for the transfer of proteins, lipids, and RNA, facilitating communication between cells (Azar et al., 2021). SCD-Exos refer to exosomes carrying specific proteins and nucleic acids associated with stem cell functions. Unlike stem cells, exosomes cannot replicate, eliminating concerns about potential tumorigenesis after transplantation. In HF, exosomes are sourced from various cells, including DPCs and MSCs (Azar et al., 2021).

DPCs-Exos can regulate key signaling pathways like Wnt/β-catenin and BMP, promoting HFSC proliferation, regeneration, and HF formation. MSC-derived exosomes activate Akt signaling and increase the anti-apoptotic protein Bcl-2 in DPCs, transitioning HFs from a dormant to an active phase. While animal studies have been encouraging with minimal side effects, further clinical research is needed to understand their specific effects, mechanisms, and potential risks in the human body (Salhab et al., 2022).

Overall, this part provides a narrative overview of currently utilized pharmaceutical agents and selected prospective therapeutic approaches for addressing hair aging. It is evident that the existing therapeutic strategies for hair loss treatment have failed to produce satisfactory outcomes with significant adverse effects. The mechanisms are also poorly understood. The etiology of hair loss, substantial inter-individual variability, and disparities between animal models and humans in manifestation and therapeutic efficacy have resulted in inconsistent conclusions. Methodological flaws have further hampered the reliability and comparability of the data. Nevertheless, multiple new pharmacological targets have been employed in drug discovery, including JAK inhibitors and PGD inhibitors. Among which, baricitinib has recently been approved for the treatment of AA. It is hoped that as the understanding of these mechanisms becomes more comprehensive, more potent treatments will be developed to tackle the hair aging problem.

## Discussion

In our review, we explored many complex factors responsible for HF aging. The discussion highlighted mechanisms associated with the aging process.

Firstly, we emphasized the role of oxidative stress, which is mainly mediated by ROS. The oxidative stress might cause DNA damage, lipid peroxidation, and long-term inflammation, all of which contribute to hair aging. Furthermore, the mechanisms regarding how HFPU responds to oxidative stress and leads to hair graying have also been clarified.

Additionally, discussions about hormonal influences on hair aging focus largely on androgen and estrogen. Androgens, particularly DHT, have a substantial impact on HFs, contributing to diseases such as MPHL. Estrogens, on the other hand, play a crucial role in promoting HF growth as well as protecting HFs from antioxidant damage. Chronic stress-induced hormones such as cortisol also account for hair aging.

Besides, we conclude that hair aging can be triggered by inflammatory signaling. Among these immune disorders, the activation of JAK-STAT signaling is observed in multiple premature hair aging diseases such as AA, AGA, and cicatricial alopecia. Previously, JAK-STAT was known to be involved in immune response, tissue regeneration, and apoptosis (Hu X. et al., 2021). Given the critical role of JAK-STAT signaling in maintaining immune functions, it has great potential for inflammation-driven alopecia treatment. For example, JAK inhibitors are reported to alleviate the AA symptoms, probably by reducing cytotoxic T cells and IFN-γ signature in HFs (Harel et al., 2015). Moreover, stress-induced neurogenic inflammation is drawing people’s attention. Nowadays, more and more people are suffering from psychological diseases such as anxiety and depression, which often occur with premature hair graying and hair loss. Several studies have uncovered the correlation between perceived stress and HF disorders (Liu et al., 2013; Zhang et al., 2020; O’Sullivan et al., 2022). Therefore, stress-associated neurotransmitters such as norepinephrine might also participate in the aging process, which is still largely under-explored. In addition to that, a recent study has shown a microbiome discrepancy in aged and young scalp skin. Among these microbiomes, *Acinetobacter* was significantly increased in aged scalp skin, and further research is required to confirm its role in hair aging (Shibagaki et al., 2017). How other bacteria relate to hair aging, such as *Malassezia*, *Staphylococcus* spp., and *Alternaria* spp., is also worth investigating due to their potential ability to cause scalp diseases and HF disorders (Polak-Witka et al., 2020). Moreover, the interaction between these microbiomes and the innate immune system remains unclear, which is under extensive research (Sakamoto et al., 2021).

HF homeostasis is regulated by intrinsic and extrinsic signaling, emphasizing the role of the HF milieu in hair aging. For instance, recent research has demonstrated that lymphatic vessels are important for skin renewal (Gur-Cohen et al., 2019; Peña-Jimenez et al., 2019). The size of SGs also changes over time during aging, along with altered sebum composition (Hou et al., 2022). Additionally, dermal white adipose tissue (dWAT) became thicker with age, accompanied by aberrant inflammatory signaling (Chen et al., 2021). Sympathetic nerves within the HF regulate HFSC activation while depleting McSCs in HFs (Shwar et al., 2020; Zhang et al., 2020). These findings demonstrate that the HF milieu might be important for the hair aging process.

In conclusion, hair aging is a complex process influenced by numerous factors. In our review, we examine the features of hair aging, including hair graying, baldness, and HFM. These processes can be attributable to oxidative stress, hormonal imbalance, inflammation, and DNA damage. Understanding the complex mechanisms of hair aging and emerging trends in treatments helps us develop effective therapeutic strategies. With more and more in-depth research in this field, we anticipate further breakthroughs in hair aging mechanisms and treatments.
